# Exercise in space: the European Space Agency approach to in-flight exercise countermeasures for long-duration missions on ISS

**DOI:** 10.1186/s13728-016-0050-4

**Published:** 2016-08-02

**Authors:** Nora Petersen, Patrick Jaekel, Andre Rosenberger, Tobias Weber, Jonathan Scott, Filippo Castrucci, Gunda Lambrecht, Lori Ploutz-Snyder, Volker Damann, Inessa Kozlovskaya, Joachim Mester

**Affiliations:** 1 Wyle GmbH, Cologne, Germany; 2 Space Medicine Office (HSO-AM), European Astronaut Centre Department, Directorate of Human Spaceflight and Operations (D/HSO), European Space Agency (ESA), Geb. 12, Linder Höhe, PO Box 906096, 51147 Cologne, Germany; 3 Deutsches Zentrum fuer Luft-und Raumfahrt, Cologne, Germany; 4 Universities Space Research Association, NASA Johnson Space Center, B261, SK3, Houston, TX 77058 USA; 5 Institute for Biomedical Problems (IBMP), Russian Space Federation/Roscosmos, Khoroshevskoe Shosse, 76A, 123007 Moscow, Russia; 6 Institute of Training Science and Sport Informatics, German Sport University Cologne (DSHS), Am Sportpark Muengersdorf 6, 50933 Cologne, Germany; 7 International Space University (ISU), Parc d’Innovation, 1 Rue Jean-Dominique Cassini, 67400 Illkirch-Graffenstaden, France

**Keywords:** Exercise countermeasures, Microgravity, European Space Agency, International Space Station, Astronaut training, Space flight, Physical performance

## Abstract

**Background:**

To counteract microgravity (µG)-induced adaptation, European Space Agency (ESA) astronauts on long-duration missions (LDMs) to the International Space Station (ISS) perform a daily physical exercise countermeasure program. Since the first ESA crewmember completed an LDM in 2006, the ESA countermeasure program has strived to provide efficient protection against decreases in body mass, muscle strength, bone mass, and aerobic capacity within the operational constraints of the ISS environment and the changing availability of on-board exercise devices. The purpose of this paper is to provide a description of ESA’s individualised approach to in-flight exercise countermeasures and an up-to-date picture of how exercise is used to counteract physiological changes resulting from µG-induced adaptation. Changes in the absolute workload for resistive exercise, treadmill running and cycle ergometry throughout ESA’s eight LDMs are also presented, and aspects of pre-flight physical preparation and post-flight reconditioning outlined.

**Results:**

With the introduction of the advanced resistive exercise device (ARED) in 2009, the relative contribution of resistance exercise to total in-flight exercise increased (33–46 %), whilst treadmill running (42–33 %) and cycle ergometry (26–20 %) decreased. All eight ESA crewmembers increased their in-flight absolute workload during their LDMs for resistance exercise and treadmill running (running speed and vertical loading through the harness), while cycle ergometer workload was unchanged across missions.

**Conclusion:**

Increased or unchanged absolute exercise workloads in-flight would appear contradictory to typical post-flight reductions in muscle mass and strength, and cardiovascular capacity following LDMs. However, increased absolute in-flight workloads are not directly linked to changes in exercise capacity as they likely also reflect the planned, conservative loading early in the mission to allow adaption to µG exercise, including personal comfort issues with novel exercise hardware (e.g. the treadmill harness). Inconsistency in hardware and individualised support concepts across time limit the comparability of results from different crewmembers, and questions regarding the difference between cycling and running in µG versus identical exercise here on Earth, and other factors that might influence in-flight exercise performance, still require further investigation.

## Background

On April 12, 1961, aboard Vostok 1, Yuri Gagarin completed a single orbit of the Earth and, in the process, achieved the first ever human space flight. In 108 min between launch and landing, he spent approximately 89 min in orbit and thus also became the first human to experience a sustained period of the unique environment that is microgravity (µG). More than 50 years have passed since Gagarin’s flight, and in that time, over 500 people have flown in space, a permanent, multi-occupancy habitat in the form of the ISS has been built, and it is now routine for astronauts to participate in long-duration missions (LDM), during which they live and work in µG for periods of around 6 months.

The increase in astronaut numbers and the length of missions, and the resulting rapid increase in the total number of man-days in space—over 2000 per year on ISS—have also revealed the profound multi-system changes that take place in the human body as it adapts to µG. This adaptation, frequently referred to as de-conditioning, because the changes that occur are unfavourable for life in Earth’s gravitational environment, is associated with reductions in bone mass, muscle volume and strength, and cardiovascular capacity, and changes to blood pressure regulation and vestibular and sensorimotor function [1–7], while publically, the effects of LDMs are characterised by post-flight images of markedly weakened crewmembers struggling to walk and occasionally fainting. The recent situation is, however, considerably different.

Based on post-flight observations of the physiological adaptation to µG and the large body of knowledge concerning the effects of different types of exercise on the cardiovascular and musculoskeletal systems, great progress has been made in in-flight exercise devices and exercise program designs, which, together, have become increasingly effective in countering µG-induced adaptation. Recent data from some (but not all) ISS crewmembers, who have had access to the latest generation of devices and followed prescribed and intense training regimes during LDMs, show little or no change in bone mass [8] and cardiovascular capacity [2], while the decreases in muscular force production are becoming progressively smaller [3]. Although countermeasures have become more effective over the past decade, there are differences between ISS international partner countermeasure concepts [9]. For experiments conducted with ESA crewmembers, who serve as volunteers for the majority of ESA human physiology experiments on ISS, the influence of the ESA countermeasure program may be critical for interpreting scientific results.

The design of exercise devices for use in space must consider several important factors. First, the absence of the effects of gravity, and thus object and body weight, must be accounted for in devices designed for exercises that rely in part (e.g. weight-lifting) or entirely (e.g. running) on body weight for their exercise stimulus [9]. For a weight-lifting/resistance device, this means that the load capacity of the device must provide sufficient maximal loading for the strongest crewmembers performing exercises with a significant body weight component, such as the squat. For running exercise, the crewmember must be restrained in a manner that creates ground reaction forces comparable to running on Earth [10], but also allows for the natural rise and fall of the body’s centre of mass during the gait cycle. Second, independent of the mode of exercise, devices must be carefully isolated from the space vehicle/habitat to prevent the transmission of vibration, forces, and torques to the spacecraft structure [9, 10]. The design of the exercise devices currently available on ISS in 2015 reflects the principles described above. Although owned and operated by either the National Aeronautics and Space Administration (NASA) or The Russian Federal Space Agency (Roscosmos), they are accessible to crewmembers from the other ISS International Partners: ESA, the Canadian Space Agency (CSA), and Japan Aerospace Exploration Agency (JAXA).

The JAXA and CSA have initially elected to have their crewmembers follow in-flight exercise programmes provided by NASA, although to a different extent. In 2004, ESA’s Space Medicine Office embarked on the development of its own physical exercise concept for crewmembers, including an in-flight countermeasure program for LDMs. Albeit bearing many similarities to the concepts used by the other ISS partners, it has a number of conceptual differences, some of which are a result of the unique conditions under which ESA astronauts and the Space Medicine Office Exercise Specialists must operate. Established in 2006 and subsequently refined, ESA’s exercise support concept and in-flight exercise countermeasure regime have now been used for the LDMs to ISS of eight ESA crewmembers. In the 10 years since, human space flight has undergone considerable change: relatively simple exercise devices have been replaced by more advanced ones with increased functionality [11], exercise prescriptions for use of these devices have developed and optimised [9], and the marked physiological deterioration that is historically associated with time in µG has been replaced by crewmembers returning in remarkably good physical condition [1, 2, 8]. As such, now is an appropriate time to summarise and review the implementation and outcomes of ESA’s in-flight exercise countermeasure program for LDM crewmembers living and working on ISS.

The purpose of this paper is, therefore, threefold: first, it is to provide a description of the ESA Space Medicine Office’s individualised approach to the delivery of exercise support to ESA crewmembers, particularly the provision of in-flight exercise countermeasures during LDMs; second, it is to provide the reader with an up-to-date picture of the human spaceflight on the ISS and how exercise is used to counteract physiological changes resulting from adaptation to prolonged exposure to µG; and finally, it is hoped that it will serve as a reference document for scientists planning and implementing experiments on ISS in which ESA crewmembers serve as volunteers, to aid them in both interpreting their data and designing future experimental protocols that are compatible with ESA medical operations requirements.

## Methods

### Ethics approval

In accordance with the North Rhine (Germany) Medical Association’s professional code of conduct (§ 15 BO), as this study was a retrospective analysis of data collected as part of standard ESA medical monitoring and all crewmembers remained anonymous, no specific ethical approval was required nor was the written consent of the crewmembers who participated in the missions analysed (Communication Reference Number 42-2016 from the North Rhine (Germany) Medical Association’s Ethics Board).

### European astronauts

The current ESA Astronaut Corps comprises individuals from two separate selection processes, one during the years 1998–2000, during which ten astronauts were recruited, and the second in 2008–2009, when further six were selected. All successful candidates met basic physical selection requirements, which included having a stature of between 149.5 and 190.5 cm, and a body mass of <95 kg, and subsequently passed a rigorous medical and psychological assessment. European LDMs (greater than 30 days and up to approximately 6 months) to ISS were first conducted in 2006, and at the time of writing (May 2016), eight missions have been completed. For all ESA missions between 2006 and 2015, the mean and standard deviation (±SD) duration was 162 ± 48 days (range: 49–200 days). The characteristics of the eight crewmembers who completed these missions are shown in Table 1.

**Table 1:** Characteristics of astronauts (n = 8) who have completed the European Space Agency’s eight long-duration missions (LDMs) to the International Space Station (ISS)

Measure	Mean	SD	Minimum	Maximum
Age at selection in (years)	36	4	31	41
Age at first space mission (years)	40	6	31	50
Age at time of ISS LDM mission (years)	45	7	37	54
Time from selection to ISS LDM (years)	7	3	4	12
Stature at ISS LDM launch (m)	1.80	0.09	1.65	1.89
Body Mass at ISS LDM launch (kg)	80.5	11.7	62	95
ISS LDM duration (days)	163	48	49	200
Total time in space (days)	191	73	69	350

*SD* 1 standard deviation

### Individualised exercise training approach for ESA astronauts

The program for ESA crewmembers is an individually tailored approach in which ESA’s exercise specialist considers individual fitness levels, personal preferences, and career status, as well as ISS exercise hardware specifications. Required crew health standards are outlined in the operational medical evaluation document [12], and include all physical assessments performed annually and before, during and after missions.

Throughout their active career (when they are not completing or recovering from a mission), ESA astronauts are required to maintain an above average level of physical fitness using self-guided exercise programmes that are supported as required by an ESA exercise specialist. The requirement for a high level of autonomy in terms of adhering to regular physical activity is a consequence of the ESA-specific work conditions, in which ESA astronauts spend the majority of their mission preparation time away from their duty home base, the European Astronaut Centre (EAC) in Cologne, Germany. This results in limited direct contact, with crew supported remotely by the exercise specialists and locally by specialists from the other ISS partners. This is in contrast to NASA astronauts and Roscosmos cosmonauts who can spend a much greater proportion of their time with their own agencies’ physical exercise specialists at their home base.

Exercise activities are planned in several phases with specific characteristics and objectives (e.g. mission assignment vs no mission assignment), whereas the in-flight phase, involving daily prescribed exercise, is the most compact and intense exercise period. A unique phase during the astronaut career is the so called “Basic Training”, which occurs once in the time period shortly after selection as astronaut candidates and lasts approximately 1 year. During this time, ESA astronauts develop basic exercise skills (e.g. the correct performance of free-weight lifting exercises and the operation of exercise hardware, such as heart rate monitors, and the preparation for exercise following ISS protocols) in dedicated one-to-one sessions with the exercise specialists, and are encouraged to participate in a broader range of physical activities and sports. After basic training, more remote support concepts are applied to accompany the astronauts during training phases and mission preparation.

### Pre-flight exercise

The main objective of pre-flight (and general) exercise training is to support astronauts in maintaining an overall fitness level that is above average for their age [13]. In the pre-flight phase, which begins with mission assignment between one and 2 years prior to launch, the exercise program consists of a mix of supervised (with either the ESA exercise specialists at EAC or the local exercise specialist if crew is training elsewhere) and unsupervised exercise sessions. The supervised sessions consist of typical gym exercises, but with specific focus on the development and implementation of an individualised ISS in-flight exercise protocol. Training with replicas of station-specific “flight-like” countermeasure devices (see “In-flight exercise hardware” section below) is led by dedicated device experts, supported by ESA exercise specialists. On a regular (at best monthly) basis, astronauts are requested to provide their training data, including the types of exercises performed, and training time and intensity (heart rate or subjective intensity), and their personal feedback. These data, in conjunction with the results from standardised pre-flight tests (see “Astronaut fitness evaluation” section below), are used by the exercise specialists to individually tailor the ISS exercise countermeasure training protocols for each astronaut prior to the mission starting.

### In-flight exercise hardware

The countermeasure exercise devices on ISS available during ESA LDMs have varied over time (Table 2). For cardiovascular exercise, two cycle ergometers have been available: the cycle ergometer with vibration isolation and stabilisation (CEVIS), providing workloads from 25–350 Watts, and VELO (100–250 Watts); treadmill running was performed by USOS (United States On-orbit Segment—which includes ESA astronauts) crew on the treadmill with vibration isolation and stabilisation system (TVIS) (providing motorised speedup to 16 km h^−1^) until 2009 and, subsequently, on the 2nd generation treadmill (called COLBERT or T2) (providing motorised speedup to 20.4 km h^−1^) and the BD-2 treadmill (up to 20 km h^−1^) [9, 11]. ‘Passive’ modes can also be used, where crewmembers are required to move the belt themselves. Resistance exercise was performed on the interim resistive exercise device (iRED) (providing load from 5 to 136 kg) until 2009 and, subsequently, on ARED (providing loads from 2.2 to 272 kg) [9, 14, 15]. The VELO device also had “force loaders”, motor-driven cords that provided loads of up to 30 kg, attached to it [16]. Throughout all missions, access to the different hardware types for ESA crewmembers was not consistent, both for technical (mechanical) and organisational reasons (US and Russian hardware are administratively separated, and ESA crewmembers were affiliated to either US or Russian crews).

**Table 2:** Historical overview of exercise countermeasure hardware available on ISS for ESA’s eight long-duration missions to the International Space Station (ISS)

Year	Hardware used by ESA crew on ISS	ESA mission
2000–2009	Treadmill (TVIS, BD-1)	LDM 1–3
2000–2009	Resistive exercise device (iRED)	LDM 1–3
2009–	Treadmill (T2), resistive exercise device (ARED)	LDM 3–8
2013–	Treadmill BD-2	LDM 6–8
2001–	Cycle ergometer (CEVIS, VELO)	LDM 1–8

*iRED* interim resistive exercise device; *ARED* advanced resistive exercise device; *TVIS* treadmill with vibration isolation and stabilisation system; *T2* 2nd generation treadmill; *BD*-*1/, BD*-*2* (Roscosmos) “Begushaya Dorozhka 1/2”; *CEVIS* cycle ergometer with vibration isolation and stabilisation system; *VELO* Russian cycle ergometer

### In-flight exercise countermeasures

The in-flight, individualised training approach with ESA crewmembers includes three phases [17] (Table 3). The duration of each phase is varied depending on the length of the mission, as well as individual crewmember-specific factors including their adaptation to µG and exercise on ISS exercise hardware, and their individual training response to in-flight exercise. Typically, for in-flight exercise, an adaptation time of 2–3 weeks is scheduled (referred to as the Adaptation Phase or Phase 1), with the first exercise sessions planned for the cycle ergometer. The first scheduled exercise bout (of a maximum of 1 h) is conducted no earlier than the second day after arrival on ISS, and this is followed by an increase in exercise time and loading up to the scheduled 2.5 h. The use of cycle ergometer, treadmill, and resistive exercise devices is relatively balanced in this phase, with 4–5 sessions per device each week in a periodic order. The intensity of the initial sessions is relatively low (e.g. 50–60 % of pre-flight capacity established in pre-flight training sessions) and increased subsequently per crew discretion.

**Table 3:** The three in-flight phases of ESA’s personalised training approach for long-duration mission crewmembers

Phase	Phase name and purpose	Duration (nominal)
1	*Adaptation phase*—familiarise crewmember with ISS exercise hardware and adapt the crewmember to exercise in microgravity	First 1–20 days of mission
2	*Main phase*—prevent physiological adaptation to microgravity	Approximately 130–150 days
3	*Preparation for Re*-*entry* *phase*—prepare crewmember for rigours of re-entry and potential off-nominal landing scenarios	Final 15–30 days of mission

For the ‘Main’ Phase (Phase 2), training loads for resistance exercise are increased at a rate of 3–5 % per week, while the rate of increase in treadmill load (speed and vertical loading through harness) and cycle ergometer (workload) is less structured and incudes periodic increases based on crewmember performance. Training loads are targeted toward 80 % or higher of individual maximal capacity [18] established in pre-flight training and testing sessions, but also adapted to the individual. The vertical loading for treadmill running provided by the harness as an additional training parameter is measured statically by force sensors integrated into the treadmill surface (or by loading calculations provided by NASA [19] before this technology was availableconsidering crew height and body mass. Loading is set to approximately 50 % of body weight for the first 1–2 weeks in Phase 1 and then gradually increased during Phase 2. Typically, the maximum that can be achieved comfortably is 70–80 % of body weight, with the maximal load depending on crewmember height and body weight, the restraint system used (elastic bungee cords [19] were used for most ESA LDMs) and individual tolerance of the discomfort that can occur at higher levels of loading.

In the ’Preparation for Re-entry’ Phase (Phase 3) during the last 3–4 weeks on ISS, training loads are kept high, with an increasing focus on resistive exercise and treadmill running and the elimination of cycle ergometry (Table 3). If possible, further increases in load are implemented, whilst ensuring good posture control during resistance training to avoid injury.

The nominal scheduled in-flight exercise time allowance for all astronauts is 2.5 h per day, including setup, stow, and personal hygiene. As such, the actual time spent exercising is approximately 1.5 h per day. For European astronauts, exercise is prescribed 7 days per week, with the goal of achieving a total of 6–7 resistance and 4–7 cardiovascular sessions per week, adapted to crewmember preference and based on the ESA countermeasure concept. Daily exercise consists of one bout of resistance and one bout of cardiovascular (either treadmill or cycle ergometer) exercise, either back to back or split up into two separate sessions, per crew preference. As the muscles and bones of the lower limbs are most sensitive to µG adaptation, the main resistive exercises prescribed are squats, heel raises. and deadlifts, and are performed during every session with minor variations (e.g. sumo squats). To provide variety for the crewmember and to ensure a comprehensive whole-body work-out, a range of other resistive exercises, such as crunches and bench presses [20], are included and varied from session to session. Depending on the protocol, the number of repetitions ranges from 6 to 15, and the number of sets ranges from 2 to 5 (Table 4). Pre-flight performance and personal feedback from the crewmembers during the mission are also used for updating exercise prescriptions and modifications to the exercise program.

**Table 4:** Outline of ESA in-flight exercise protocols for long-duration missions (LDM) utilising International Space Station (ISS) exercise hardware

	Resistive protocols	Treadmill protocols	Cycler ergometer protocols
Type	Interval (6,8,15 reps, 3–5 sets, daily rotation)	Continuous Interval Slope Individual	Continuous Interval Slope Hill
Load	Variation of load and repetition (6–15 reps, 3–5 sets)	Low/60 %, medium/75 %, high/85 %^a^ (alternating daily)	Low/60 %, medium/75 %, high/85 %^a^ (alternating daily)
Progression	3–5 %/wk (upper and lower limbs) *Phase 1*: initially ^a^lower/50–60 % loads, CM increases load at discretion *Phase 2*: systematic increase based on Phase 1 final load *Phase 3*: maintain high loading or increase (3–5 %)	Between 0 and 5 speed (km/h) or interval duration (min) increase events over mission *Phase 1*: Initially lower loads, CM increases load at discretion *Phase 2*: Individual increase (SLS load [kg], intervals [min]/number, speed [km/h], protocol type based on crew feedback *Phase 3*: continued increase in load (if possible)	Initial workload (W) decrease up to −30 %, increase to 100 % toward end of mission *Phase 1*: Initially lower loads, CM increases load at discretion *Phase 2*: Individual increase of workload (W), intervals (min)/number, protocol “shape” based on crew feedback *Phase 3*: continued increase (if possible)
Duration (average)	60 min	30 min	30 min

*CM* crewmember; *CMS* countermeasures; *IRED* interim resistive exercise device (LDM 1 + 2); *ARED* advanced resistive exercise device (LDM 3–8); *TVIS* treadmill with vibration isolation and stabilisation system (LDM 1–3); *T2* 2nd generation treadmill (LDM 3–8); *CEVIS* cycle ergometer with vibration isolation and stabilisation system (LDM 1–8); *VELO* Russian cycle ergometer; *SLS* subject loading system

^a^Intensity/ % relative to individual’s maximal capacity

### In-flight exercise constraints

There are multiple factors affecting the exercise countermeasure program [9, 16, 21]. For example, astronauts performing extravehicular activities (EVAs) do not exercise on that day and some human physiology experiments in which crewmembers are participating in record parameters that are affected by exercise, resulting in exercise program restrictions, including cancellation of sessions or limitations on exercise intensity. There are also exercise constraints associated with visiting vehicle docking and undocking events, engine firing for reboosting the station to a higher orbit, and robotic arm operations. The use of iRED, which was hard mounted to the ISS structure—and thus transmitting dampened loads to the structure with some exercises—was limited during LDM 1 and 2 (2006, 2008) to protect the station and maintain hatch sealing (Personal Communication, ESA Biomedical Engineer). High atmospheric CO_2_ levels (>7 mmHg) can lead to exercise restrictions to prevent further increases, and any emergency situation (e.g. fire, reduction in cabin pressure drop, potential air toxicity), crew sleep shifts and individual crew health issues (injury and sickness) may all lead to operational constraints that require alternative exercise plans. In the case of hardware failures, “back-up hardware” can be used in a so-called “contingency mode” (e.g. BD-2 in place of T2 for treadmill running for USOS crewmembers) to minimise the effects on the overall ISS exercise program. Presently, if ARED were to become unavailable, with iRED no longer on ISS, resistive exercise can only be performed using the force loader on VELO and rubber/Thera bands. If one of the two treadmills were to become unavailable, all crewmembers would use the remaining device, but long-term failure might also result in evacuation. For cardiovascular training, treadmill exercise is considered a suitable surrogate for cycle ergometer training, but not vice versa, as cycle ergometry is considered less functionally relevant for return into Earth’s gravity. Finally, exercise sessions are voluntary (although not the countermeasure training as a whole), and each crewmember retains the right to opt out of any individual exercise session and may do so in coordination with their assigned exercise specialist.

### In-flight monitoring of exercise

Exercise training on ISS involves significantly more time and effort than on Earth and requires close health supervision. Heart rate during cardiovascular exercise sessions is monitored using a chest strap (Polar, Kempele, Finland), and heart rate and workload data files are stored directly on ISS computers and downloaded once per week by the hardware owner (NASA) and reviewed by the ESA exercise specialist. As a contingency for the failure of heart rate data storage, wrist-worn receivers are available, but the data must be downloaded manually by the crewmember. Cardiovascular performance is monitored during periodic fitness evaluations (PFE) to estimate maximal oxygen uptake (VO_2max_—based on a submaximal [25–75 % VO_2max_] standardised protocol), starting on Flight Day 15 and then monthly throughout the mission [2]. In LDM 1 and 2, an additional treadmill and cycle ergometer test was conducted using the Roscosmos Russian Medical Operations “MO-3” and “MO-5” [16] [22] protocol, but this was discontinued in 2009. For resistive exercise, the total number of repetitions and sets, and the load used are recorded by the crewmember on an ISS computer. An automated data capturing system for ARED is planned with the goal of being operational in 2016 (Personal Communication, NASA Engineering). Data files are downloaded once per week by the hardware owners (NASA) and reviewed by an ESA exercise specialist. Following the introduction of ARED on ISS, with its ability to deliver higher loads than iRED, three privatised, real-time audio and video coaching sessions are conducted during an ISS mission. Feedback to the crewmember is provided by the ESA exercise specialist and physiotherapist to ensure the correct lifting technique, particularly for exercises involving high loads and higher risk postures (e.g. deadlift). Two of these sessions are conducted early in the mission when the crewmembers are in the process of familiarising themselves with ARED exercise and the third later in the mission when crewmembers start to lift heavier loads. Finally, the ESA exercise specialist convenes once monthly periodic exercise conference (PEC) with the crewmember to review all aspects of the previous month’s exercise activities and agree on a plan for the month ahead.

### Data processing

For data analysis at EAC, Microsoft Excel 2010 (Version 14.0.7153.5000, Microsoft, Redmond, USA) and a statistics program (PASW Statistics 18, IBM Corporation, Armonk, USA) are used. Protocol prescriptions are also prepared with Microsoft Excel, and for T2, CEVIS, and ARED, a special NASA protocol prescription application (“CMS app”) has been used since 2011 to generate and upload protocols.

### Astronaut fitness evaluation and monitoring

Physical assessments, including the European astronaut fitness assessment [23], are performed within a set of ISS crew-specific medical assessments approximately 90–60 days before launch, and at 4–6 and 21 days after landing (Table 5). The results of the pre-flight and the first post-flight tests are used to detect spaceflight-induced changes, and the results of the two post-flight tests are used to verify the efficiency of the post-flight reconditioning program and to detect potential long-term changes in physical performance. Functional fitness assessments are also conducted by other space agencies on their crewmembers, e.g. NASA [15]. Furthermore, separate isokinetic muscle strength and neurovestibular posture testing are performed as part of the extensive medical examinations for all ISS crewmembers.

**Table 5:** Pre-, in- and post-flight fitness tests conducted with ESA long-duration mission crewmembers

Timing	Measure (Test)
Annually, and L−90,L−60, R + 4-6, R + 21	Height Body mass Body composition (bio impedance) Flexibility (Sit and reach, Thomas test Postural stability (Pressure plate and balance board) Hand grip strength Muscle power (Squat, countermovement and drops jumps) Major muscle group strength (1RM bench press, squat) Core muscle endurance (time to exhaustion) Cardiovascular capacity, LT and IAT (modified Bruce treadmill protocol)
L−300, L−90, L−60, R + 4-6, R + 21	Muscle strength (Isokinetic)
L−300, L−90, L−60, in-flight (FD15 and then every 30 days), R + 4-6, R + 21	Spiroergomtery (100 % [pre- and post-flight only], 25–75 % cardiovascular capacity on cycle ergometer)

*1 RM* One repetition maximum; *LT* lactate threshold; *IAT* Individual anaerobic threshold; *FD* flight day (on ISS); *L*– launch date minus (number of days); *R*+ Return date plus (number of days)

### Post-flight exercise reconditioning

Within 1 day of landing, a 21-day post-flight reconditioning programme is implemented with the goal of correcting any residual performance changes due to µG adaptation and re-adapting to life in Earth’s gravity. Details of this programme will be presented in a future publication but, briefly, the programme is divided into three phases: the first is initiated by ESA’s physiotherapist, who focuses on movement quality through motor control training, and stabilisation and strengthening, utilising a variety of physiotherapy-based strategies to assess and, if necessary, make corrections. This phase transitions seamlessly into a physical exercise training program provided by the ESA exercise specialists jointly with the physiotherapist in the following phases, with the objective of fully restoring cardiovascular, musculoskeletal, and neuromuscular function to at least that of pre-flight. In addition, the ESA physiotherapist also conducts specific assessments (e.g. through manual therapy and ultrasound measurements) of function and progression during the first phase of the reconditioning programme (publication in preparation).

### Statistical analysis

The data presented below were collected from in-flight exercise data received from each ESA LDM crewmember and also from mission specific reports. Data are presented as mean (n = 8) and standard deviation (SD) for each parameter, displayed in whisker plots (with median line in the graph). For resistance exercise, Student’s *t* test for paired data was used to test for differences between the initial loads (kg) implemented with six or eight repetitions (“high” loads) during Phase 2 (Main Phase), and the final load used at the end of Phase 3 (Preparation for Re-entry Phase). *Student’s* t-test for paired data was also used to test for differences between power output (W) for cycle ergometry, and running speed (km h^−1^) and harness loading (% of body weight) from the same time periods.

## Results

The total number of sessions completed on all ISS exercise devices by the eight LDM ESA crewmembers was 1785. Six different (Roscosmos and NASA) countermeasure devices were used in this time period, providing resistive, treadmill, and cycle ergometer exercise. The mean (±SD) number of sessions per device for all missions was 98 ± 45 for resistive exercise, 79 ± 41 for treadmill running, and 48 ± 23 for cycle ergometry. Across all eight missions, 44 % of exercise sessions were resistive exercise, 35 % treadmill running, and 21 % cycle ergometry sessions, resulting in a balance of 44–56 % between resistive and cardiovascular exercise.

### Pre- and post-“ARED era” exercise sessions

**Fig. 1:**
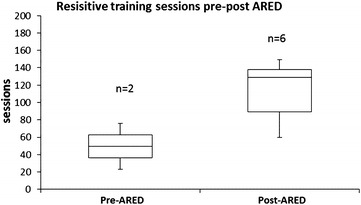
The total number of resistive exercise sessions performed per mission prior to, and following, the installation of the advanced resistive exercise device ARED installation on ISS. Pre, prior to ARED installation (long-duration missions 1 and 2); Post, following ARED installation (long-duration missions 3–8). The *median line* is indicated in the *boxplots*

As the total number of resistance exercise sessions increased markedly after the installation of ARED (between LDM 2 and 3), the influence of ARED on the in-flight exercise program is presented in relation to other exercises performed. Comparing resistive and cardiovascular exercise sessions before and after the installation of ARED (LDM 1 + 2 vs LDM 3–8), the contribution of resistive exercise sessions increased from 33 to 46 % (Fig. 1), whilst the contribution of cardiovascular sessions decreased from 67 to 54 %. Comparing LDM 1 + 2 and LDM 6–8, the contribution of treadmill sessions decreased from 42 to 33 % and cycle ergometry from 26 to 20 %. There was no evidence of increased or decreased treadmill running associated with the exchange of the treadmills (TVIS to T2 between LDM3 and 4 in 2009).

### Workload progression

A comparison of the loads used in the first exercise session of Phase 2 with those used in the final sessions before the end of the mission (Phase 3) showed significant increases for the majority of parameters measured.

**Fig. 2:**
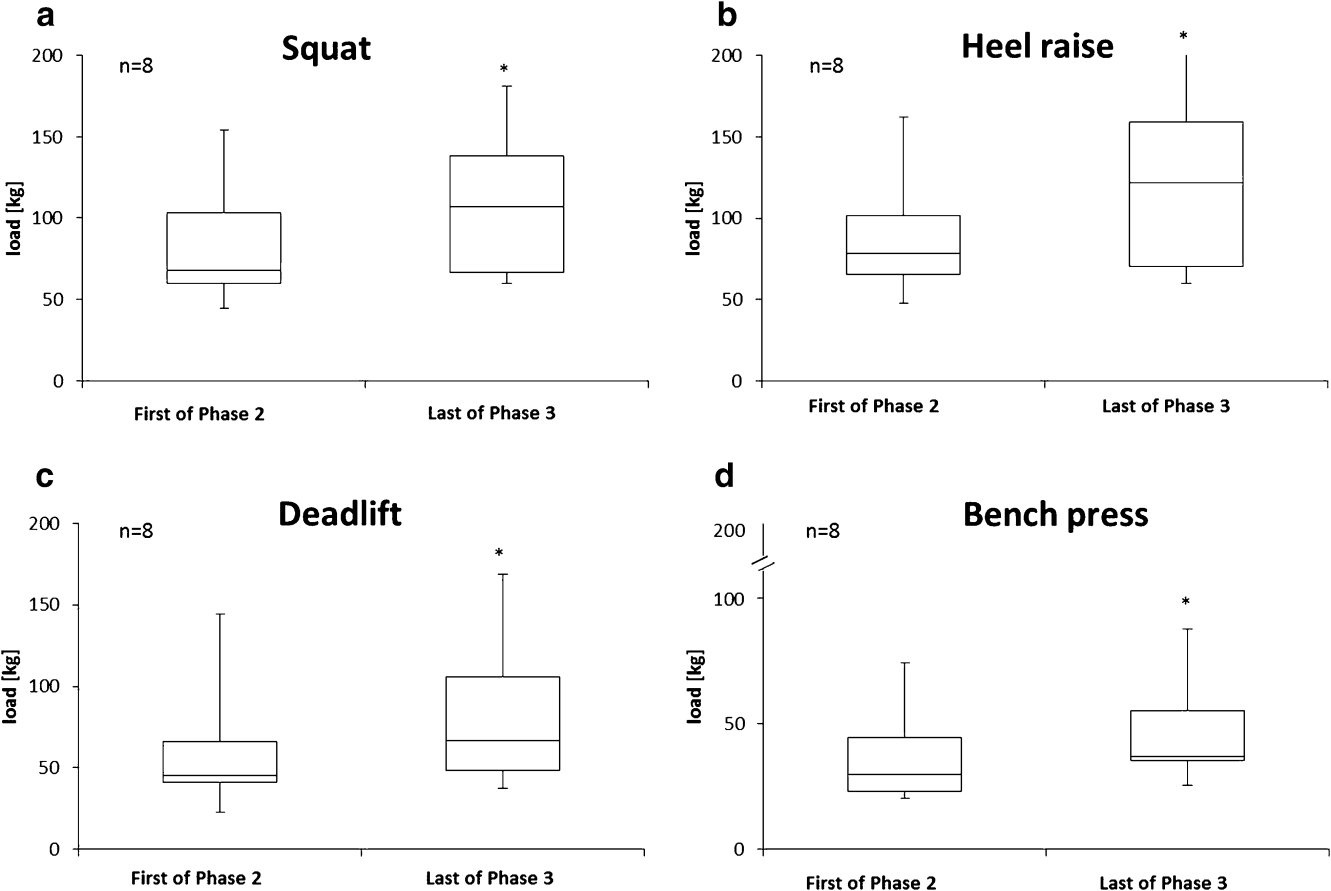
Workload (n = 8) during the first resistive exercise session of Phase 2 and the last session of Phase 3 (and of the mission) for squats (**a**), heel raises (**b**), deadlifts (**c**), and bench presses (**d**). Phase 2, Main Phase; Phase 3, Preparation for Re-entry Phase. The *median line* is indicated in the *boxplot*s. *Different (*P* ≤ 0.05) vs. the first of Phase 2

*Resistive exercise*: The progression in resistive exercise workloads is shown in Fig. 2a–d. Student’s *t* test showed significant increases for squat (*P* < 0.05), heel raises (*P* < 0.05), deadlift (*P* < 0.05), and bench press (*P* < 0.05).

### Cardiovascular exercise

**Fig. 3:**
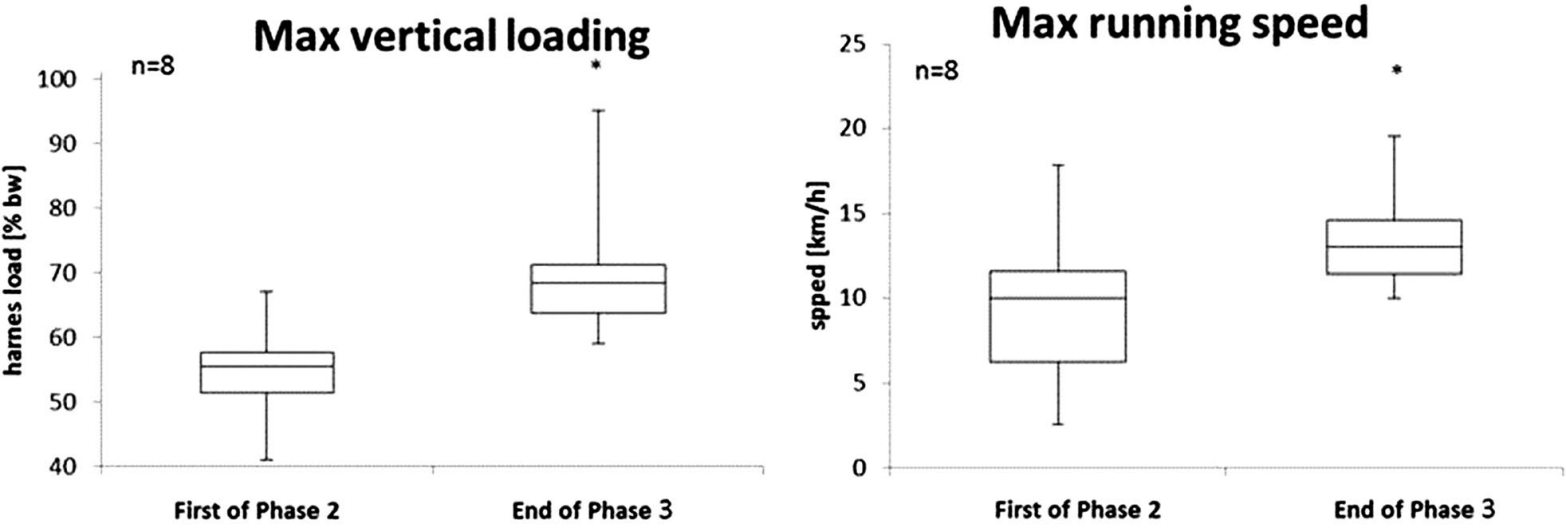
Maximum vertical loading and running speed (n = 8) during the first treadmill session of Phase 2 and the last session of Phase 3 (and of the mission). Phase 2, Main Phase; Phase 3, Preparation for Re-entry Phase. The *median line* is indicated in the *boxplots*. * Different (*P* ≤ 0.05) vs. the first of Phase 2

**Fig. 4:**
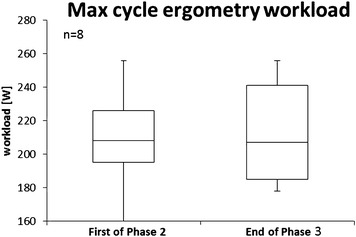
Maximum workload during the first cycle ergometry session of Phase 2 and the last session of Phase 3 (and of the mission). Phase 2, Main Phase; Phase 3, Preparation for Re-entry Phase. The *median line* is indicated in the *boxplots*. *Different (*P* ≤ 0.05) vs. the first of Phase 2

The progression in cardiovascular exercise workload from the first exercise session of Phase 2 and the final sessions of Phase 3 is shown in Fig. 3. Student’s *t* test showed significant increases in treadmill vertical loading (*P* < 0.05) and maximal running speed (*P* < 0.05), but there was no change in cycle ergometry power output (Fig. 4).

## Discussion

The present article was written to provide an update to the space life sciences and exercise community on how ESA prescribes exercise for its crewmembers before, during and after LDMs to ISS. The majority of the presented information refers to the actual in-flight phase, where astronauts exercise to minimise µG-induced changes to muscle and bone strength, cardiovascular fitness and overall health. Access to this information and its analysis is difficult for reasons that are discussed in this report, however, it may be relevant for human physiology experiments conducted on ISS utilising ESA crewmembers and measuring physiological parameters affected by exercise training.

### In-flight absolute exercise loads increase throughout LDMs

The main finding from this study is that, during the course of an LDM, crewmember absolute training loads increase rather than decrease, and they are able to maintain relative high exercise loads during prolonged exposure to µG. There are exercises (cycle ergometry) which do not show significant increases in workload despite the intention to achieve them during the mission. This may be related to both technical and biomechanical factors, as exercise in µG differs from that on Earth, with different technical and physiological constraints. Common to both parameters is a marked inter-subject variation, which may reflect the influence of both technical constraints and individual crewmember adaptation to exercise in µG.

### Low initial exercise loading the in early (Adaptation) phase of LDMs

Initially, ESA exercise specialists prescribed relatively low in-flight loading to allow crewmembers to safely adapt to exercising in µG, before systematically increasing to higher loads as the mission progresses. Starting loads are determined based on individual exercise data (heart rate and workload, resistive loads) collected in the final session before flight and are typically reduced by 10–30 % for the first in-flight sessions and progressively adapted by the crewmember during Phase 1. To support this process for resistance exercise using ARED, real-time video and audio coaching sessions are conducted to ensure correct lifting technique. In-flight load increases are, therefore, typically the greatest in Phase 1, although they are non-linear and vary considerably between crewmembers depending on individual adaptation to exercise. Once a stable basis is reached during Phase 1, systematic increases in load are applied throughout Phases 2 and 3. Although not significant for all parameters, our data show an increase in-flight absolute exercise loads for resistance exercise and treadmill running from the start of Phase 2 to the end of Phase 3 (and the mission), and, therefore, might appear contradictory to the typical post-flight performance decrements reported in the literature [2, 4, 5] after LDMs.

### Crewmembers do not exercise on the treadmill with vertical loading equivalent to full bodyweight

None of the ESA crewmembers ran on the treadmill with loading through the harness equivalent to 100 % of their bodyweight throughout their mission. Most remained at 70–80 %, which is within the typical range for ISS crewmembers [9, 19]. For treadmill exercise, vertical loading loads are provided by bungee cords attached to a body harness, which has been described previously [9]. Although there are significant load increases throughout the course of missions, only rarely (and temporarily) did crewmembers use 100 % of their pre-flight bodyweight. Running on the ISS treadmill differs in several ways from terrestrial treadmill running. On ISS, current treadmill speeds are limited between 5 and 20 km h^−1^ (3–12 m h^−1^) to protect station structure and sensitive scientific experiments from mechanically transmitted vibrations [11]. Earlier treadmills had even lower maximal speeds (16 km h^−1^ or 10 m h^−1^). Furthermore, the harness loading system that ‘pulls’ crewmembers toward the running surface leads to discomfort due to pressure on the shoulders and hips, and usually requires a number of exercise sessions before crewmembers find their individually preferred setting [9, 19]. As a result of this discomfort, astronauts rarely load the harness system to the equivalent of 100 % of their body weight on Earth. Most crewmembers exercise with static loads between 70 and 80 % body weight, with loading up to 90 % or higher reported, although this remains an exception. The space-induced weight loss of about 2 % per 100 days in μG [6, 21] is not considered in this calculation as it represents only an average and not the individual change. However, it can be assumed that for all crewmembers the relative loading increases with the loss of body mass. Once established in orbit, however, running speeds tend to be faster than those for terrestrial running, which is likely due to the lower relative vertical load. Physiologically, this may be advantageous, as higher running speeds and thus a greater number of contacts with the running surface may compensate for the lack of gravitational loading [10]. It has been suggested [10] that higher running speeds, which result in greater ground reaction forces and thus greater mechanical loads on the musculoskeletal system, may be beneficial in stimulating bone formation in µG.

### In-flight prescribed exercise loading does not increase for cycle ergometry

Cycle ergometer workload did not increase significantly during the mission, although the in-flight countermeasure plan does, in principle, include this if crewmembers are able to complete protocols easily. Cycling on CEVIS is markedly different from cycling on earth as, through the effect of Newton’s third law, the ‘weightless’ body is accelerated during every pedal down-stroke in the opposite direction. In our data, cycle ergometer training on CEVIS is the only countermeasure exercise where no increase in loading is evident during the mission. This is even evident in crewmembers with a high level of physical fitness, who have followed an extensive ground-based exercise program until shortly before launch. The CEVIS device does not have a saddle, and crewmembers are not restrained via a vertical loading system like they are on T2. Instead, they are able to restrain themselves (horizontally) via a waist/hip belt to a vertical ‘back plate’ positioned behind the crank axis, and also by holding the frame. Since 2009, they have also used cleated shoes with clipless pedals, and in 2013, based on crew feedback, hand grips were added permanently to the frame. Restraining to the back plate results in a unique cycling posture, and greater effort sometimes appears to be required by crewmembers to execute protocols on the device. Some crewmembers report needing to actively pull their body towards the pedals (personal communication), causing them to become exhausted more quickly than when performing cycle ergometry on Earth at a similar power output. The reasons for this are not yet fully understood, but may be related to both biomechanical (resulting from the unique posture) and physiological (resulting from µG) factors, and require further investigation.

Although high-intensity exercise may well be required to meet the physical demands of returning into Earth’s gravity, there are in-flight limitations for increasing training loads beyond current values related to both technical hardware capability and discomfort associated with using it (e.g. vertical loading imposed by the T2 harness). For CEVIS exercise, crew discomfort has not been reported in relation to crew restraining themselves to the back plate, but only that, as described above, exercise can be more physically demanding that is expected based on the workloads prescribed. This issue might be related to the need to push the peddles downwards without gravitational support, which requires bracing/restraining the body to the CEVIS structure, whilst also having to the pull-up on the peddles and thus increasing the workload compared to pre-flight assessments on a terrestrial cycle ergometer. Before the availability of ARED, the capacity to provide the crew with high loads for resistive exercise throughout the entire mission was limited to iRED or simple bungee cords. The ESA countermeasure program underwent a significant change with the installation of ARED, which provided an opportunity to increase the prescription of resistive training and resulted in an associated reduction in cycle ergometer and treadmill exercise during subsequent missions. Nevertheless, post-ARED, the contribution of resistive and cardiovascular exercise sessions to the overall in-flight prescription remains relatively balanced (44 vs 56 %).

### Limitations

As a result of technical developments over the past decade, data format, quality, and completeness have changed, which have limited direct comparisons, for example, between different hardware [9, 11, 15, 16, 24]. This situation has improved with hardware developments, especially for the most recent missions. As such, ARED replaced iRED, T2 replaced TVIS, and BD-2 replaced BD-1. Older devices experienced frequent failure [22] resulting in restrictions that led to alterations to the in-flight countermeasure plan and the loss of exercise sessions, but reliability has also improved with the latest generation of hardware. The presented data is thus affected by this development process, resulting in crew performance being influenced not only by the individual response to µG and in-flight exercise, but also by technical conditions. There are several other issues that affect the comparability of data from different missions, including exercise being discontinued by crewmembers for personal reasons, or due to hardware failure. In the case of the latter, crew were required to manually report training loads, which they were not specifically trained for, nor did they receive additional time in their already busy schedule to do so. Finally, despite only a relatively low (n = 8) number of ESA LDMs, and those missions spanning a long period of time during which several exercise hardware changes occurred (i.e. TVIS and T2 for treadmill running, iRED and ARED for resistive training), all data were included in the load progression comparisons. As the maximal loading capabilities of these devices was different and crewmembers using older devices could not reach the high training loads available to crewmembers during more recent missions, ideally, these data should have been analysed separately. However, this was not possible due to the need to preserve the anonymity of the individual crewmembers.

### Summary and outlook

Despite exposure to µG and the associated degeneration of muscles and the cardiovascular system as reported in the literature, ESA’s eight ISS LDM crewmembers increased their in-flight exercise workload during their missions, with the exception of heal raises and cycle ergometry. This might indicate an improvement in-flight exercise performance, but likely also reflects, in part, the planned, conservative loading early in flight to allow adaption to µG exercise and thus should be investigated using available in-flight, and pre- and post-flight direct performance assessments. Additional factors, including comfort during exercise, hardware capabilities and mission profile, also affect the in-flight exercise program and exercise loading progression rates of individual crewmembers, and may thus indicate a link between crew performance and technical hardware capabilities. Crew performance measurements reflecting the efficiency of the in-flight countermeasure program, especially in relation to returning into Earth’s gravity, need to be analysed not only in terms of in-flight loading, but also by comparing pre- and post-mission physical performance and medical data. This will be the subject of future publications by ESA’s Space Medicine Office. The focus of this paper was to present a comprehensive overview of the in-flight countermeasure strategy applied with ESA crewmembers during ISS LDMs, with the aim of providing a reference for human physiology experiments conducted during these missions and to serve as a basis for future investigations of astronaut physical performance in µG.

## Abbreviations

AFA: astronaut fitness assessment
ARED: advanced resistive exercise device
BD-1/2: Begushaya Dorozhka 1/2, Russian ISS treadmills
CEVIS: cycle ergometer with vibration isolation and stabilisation system
COLBERT: combined operational load bearing external resistance treadmill
CMS: countermeasure exercise
CSA: Canadian Space Agency
EAC: European Astronaut Centre
ESA: European Space Agency
FSA: Federal Space Agency (Roscosmos)
iRED: interim resistive exercise device
ISS: International Space Station
JAXA: Japan Aerospace Exploration Agency
LDM: long-duration mission
NASA: National Aeronautics and Space Administration
MO-3/5: medical operations document
PFE: periodic fitness evaluation
RM: repetition maximum
TVIS: treadmill with vibration isolation and stabilisation system
T2: 2nd generation treadmill
USOS: United States On-orbit Segment
VELO: Russian cycle ergometer
VO2max: maximal oxygen uptake
µG: microgravity
